# Cell death in the developing vertebrate limb: A locally regulated mechanism contributing to musculoskeletal tissue morphogenesis and differentiation

**DOI:** 10.1002/dvdy.237

**Published:** 2020-09-02

**Authors:** Juan A. Montero, Carlos I. Lorda‐Diez, Cristina Sanchez‐Fernandez, Juan M. Hurle

**Affiliations:** ^1^ Departamento de Anatomía y Biología Celular and IDIVAL Universidad de Cantabria Santander Spain

**Keywords:** apoptosis, autophagy, cell death genes, lysosomes, programmed cell death, syndactyly

## Abstract

Our aim is to critically review current knowledge of the function and regulation of cell death in the developing limb. We provide a detailed, but short, overview of the areas of cell death observed in the developing limb, establishing their function in morphogenesis and structural development of limb tissues. We will examine the functions of this process in the formation and growth of the limb primordia, formation of cartilaginous skeleton, formation of synovial joints, and establishment of muscle bellies, tendons, and entheses. We will analyze the plasticity of the cell death program by focusing on the developmental potential of progenitors prior to death. Considering the prolonged plasticity of progenitors to escape from the death process, we will discuss a new biological perspective that explains cell death: this process, rather than secondary to a specific genetic program, is a consequence of the tissue building strategy employed by the embryo based on the formation of scaffolds that disintegrate once their associated neighboring structures differentiate.

## Key Findings

- We examine the functions of cell death in the formation and growth of the limb primordia.
- We analyze the plasticity of the cell death program by focusing on the developmental potential of progenitors prior to death.
- Considering the prolonged plasticity of progenitors to escape from the death process and the absence of defined genetic program in their regulation we propose that cell death is a consequence of the tissue building strategy employed by the embryo regulated by epigenetic factors .

## 1 INTRODUCTION

The brilliant and clear‐sighted review by Glücksmann^1^ in 1951 proposing a new interpretation of previous descriptive studies reporting the occurrence of dying cells in the tissues of vertebrate embryos is a milestone in research in this field. Prior to Glücksman's review, the presence of dead cells in embryonic tissues, identifiable mainly by the classical Feulgen nuclear staining procedure, was often considered a technical artifact, or unavoidable cell loss events occurring in tissues subjected to intense growing pressure. Glücksmann grouped the dying events into three distinct categories: “morphogenetic cell death” involved in sculpting the final shape of the embryonic growing organs; “histogenetic cell death,” accounting for the elimination of useless and abnormal cells resulting from tissue differentiation; and “phylogenetic cell death” responsible for the elimination of ancestral structures of transient utility in the embryo of most evolved species. Numerous research teams from different countries then helped expand the catalog of developmental death process, including those in vertebrate embryos, and in vegetal and invertebrate species.

Thanks to the contribution, among others, of John Saunders and John Fallon in the United States, Jean Milaire in Belgium, and Donald Ede and J Richard Hinchliffe in the UK, the developing vertebrate limb became a paradigm for studying embryonic cell death. Initial descriptions were focused mainly on the mesodermal component of the early limb primordia. These studies established a solid correlation between skeletal morphology and the patterns of cell death. Furthermore, the proposal of a distinctive and specific type of cell death in tissue remodeling, termed “apoptosis,”^2^ along with the identification of an evolutionarily conserved genetic cascade activated in the embryonic death process,^3^, ^4^ consolidated the view of embryonic cell death as a distinctive and regulated developmental process. Indeed, the degenerating limb processes were considered “active” and “genetically programmed” events, primarily responsible for the morphogenesis of the limb skeleton with major implications in teratogenesis and in the evolutionary diversification of the tetrapod limbs. However, subsequent studies accumulated evidence for the role of cell death in not only skeletogenesis, but also most, if not all, changes occurring in the tissue components of the developing limb. In addition, apoptosis has been discarded as a “unique” cell death mechanism in embryonic tissues, and the genetic regulators of cell death appeared to be related with the degenerative cascades activated in dying cells rather than being master regulators of developmental processes.^5^, ^6^ The aim of this report is to critically review current knowledge of cell death in the developing limb to propose a new perspective for the biological significance of cell death in embryonic systems.

## 2 THE CONVENTIONAL “FOUR AREAS” OF MESODERMAL CELL DEATH IN THE GROWING LIMB BUD OF AMNIOTES

**FIGURE 1:**
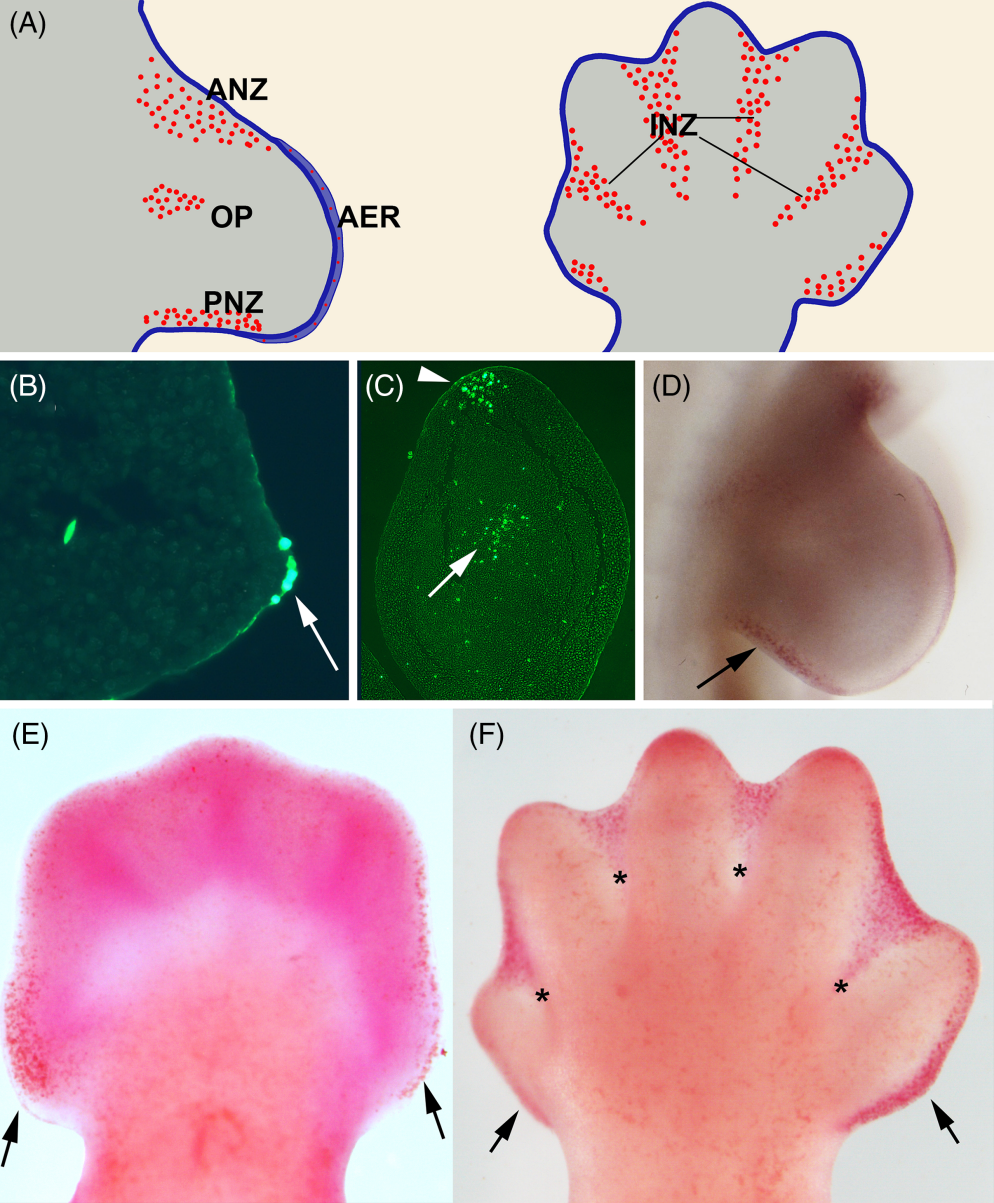
Mesodermal and ectodermal cell death in the embryonic limb. A, Schematic drawings illustrating the main areas of cell death in the developing limb (red dotted areas): the anterior necrotic zone (ANZ), the posterior necrotic zone (PNZ), the opaque patch (OP), and the interdigital necrotic zones of the mouse embryo at day 14 pc (INZ). Note also cell death scattered through the apical ectodermal ridge (AER). B, Longitudinal section of the early avian limb bud (stage HH 24) after TUNEL staining (green). Note TUNEL‐positive apoptotic cells in the AER (arrow). C, Transverse section of the avian limb bud at stage HH23, illustrating TUNEL‐positive apoptotic cells in the anterior necrotic zone (arrowhead) and the opaque patch (arrow). D, Neutral red vital staining of the chick limb bud at stage HH22 showing the posterior necrotic zone (arrow). E‐F, Neutral red vital staining of mouse autopods at day 13 (E) and 14 (F) pc. Arrows show the anterior necrotic zone and the posterior necrotic zones and asterisks the interdigital necrotic zones

The vertebrate limb primordia appear as two pairs of buds growing in the flank of the embryonic body composed of a core of mesodermal tissue covered by an ectodermal jacket (Figure 1). The employment of vital staining as a technique to map cell death in embryonic tissues allowed the detection of four characteristic areas of massive cell death in the growing limb (Figure 1A‐F). In avian embryos, these areas were termed the anterior necrotic zone (ANZ), posterior necrotic zone (PNZ), interdigital necrotic zones (INZs), and opaque patch (OP). In mouse and rat embryos (Figure 1E‐F), the mesodermal areas of cell death are similar to those of birds, but they received a different nomenclature.^7^, ^8^ As shown in Figure 1A, these areas delimit the zones of mesodermal aggregation that prefigure the skeletal pieces of the appendage. The ANZ and the PNZ eliminate the cells of the anterior and posterior margins of the bud that do not integrate into the central region where mesodermal cells form prechondrogenic condensations that prefigure the limb skeleton. The OP occupies the most central mesodermal core of the limb located between the two prechondrogenic condensations of the zeugopod (tibia/fibula; ulna/radius). INZs are by far the most prominent regions of massive cell death in the limb. These regions are located in the mesoderm intercalated between the developing digit rays, and their intensity and distribution appear closely related to the different morphology of the digits. While INZs are very prominent in mammals, birds, and reptilians, their occurrence in amphibians, both during normal development^9^ and during limb regeneration,^10^ is a more controversial question.^11^, ^12^

Comparative analysis of the patterns of these areas of mesodermal cell death in species with different limb skeletal patterns together with changes observed in mutant species with abnormal skeletal morphology have been taken as support for a sculpting function of cell death in limb morphogenesis. Multiple studies supported this hypothesis. For example, the *talpid* ^*3*^ chick mutant characterized by polydactyly showed full inhibition of PNZ and ANZ,^13^ and in turn, the *wingless* chick mutant that lacks wings showed a dramatic increase in ANZ.^14^ Similarly, the mouse *Hammertoe* mutant lacked interdigital cell death and developed soft tissue syndactyly,^15^ and the *Hemimelia‐extra toe* mouse mutant showed preaxial polydactyly associated with defective cell death in the anterior mesoderm.^16^ Furthermore, INZs have variable intensities in species with distinct degrees of interdigital webbing^17^ being very intense in species with free digits such as humans, mice, or chicks, and much less intense in species with webbed digits, such as ducks or bats.^18^ Considering this sculpting function of cell death, the differences in the pattern of cell death in a number of reptilian and mammalian species with specialized digit morphology, such as the *Chamaeleo*,^19^, ^20^ the camel, or the three‐toed rodent jerboa^21^ are particularly illustrative.

Regardless of the morphogenetic function of limb mesodermal cell death, it has been suggested that macrophages activated in the death process, generate pathways within the limb mesoderm permissive for the outgrowth of the axons that colonize the limb primordium.^22^ However, both the molecular basis and the cellular origin of the cells involved in this hypothetical function remain to be clarified.

## 3 CELL DEATH ELIMINATES EMBRYONIC FLANK MESODERMAL CELLS IN THE REGIONS THAT DO NOT FORM LIMB BUDS

As mentioned above, the limb primordia appear as two pairs of buds covered by the ectoderm that contain cells of somatopleural origin that grow in precise regions of the lateral surface of the embryonic body. An intense cell death process eliminates mesodermal cells in the interlimb region of the flank.^23^, ^24^ Notably, these cells adjacent to the limb primordia, when subjected to exogenous limb‐forming signals, escape from their dying fate, forming an otherwise normal extra limb.^25^

## 4 CELL DEATH IN THE DEVELOPING JOINTS

**FIGURE 2:**
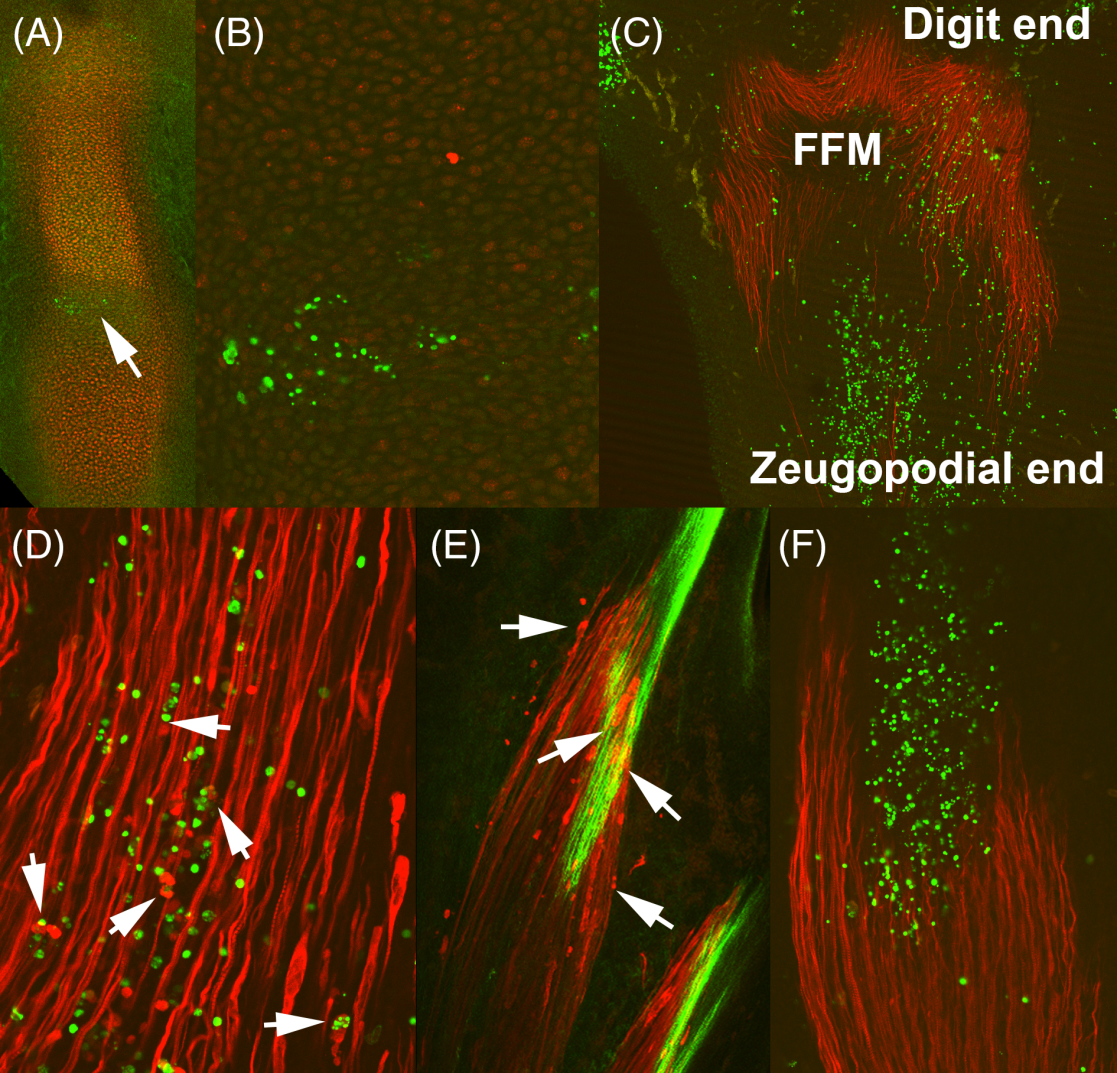
Embryonic cell death in the developing joints, muscles and tendons. A, Cell death in the joint interzone of an embryonic chick toe at stage 30 labeled for cartilage with SOX9 (red) and for cell death by TUNEL (green). Note the weakening of SOX9 expression in the developing joint area and the positive staining for cell death (arrow). B, Detailed view of the TUNEL‐positive cells in the developing joint. C‐D, Confocal images of embryonic chicken limbs at stage HH31double labeled for muscle myosin heavy chain (red) and TUNEL (green). C, Massive cell death that separates the foot flexor muscles (FFM) from the zeugopodial region. D, Detailed view of apoptotic nuclei (green) associated with muscle fibers (red). Arrows show degenerated clumped myosin associated with TUNEL positive nuclei. E, Myotendinous junction between a flexor muscle belly (red, muscle myosin immunolabeling) and its digit flexor tendon (green, tenascin immunolabeling) of a chick embryo at stage HH33. Note the condensed appearance of degenerating muscle fibers (arrows). F, Massive TUNEL‐positive apoptosis (green labeling) in a maturing myotendinous junction. Muscle myosin immunolabeling is shown in red

To form the limb skeleton, the mesodermal progenitors of the limb primordia aggregate in the core of the bud forming prechondrogenic condensations that prefigure the cartilaginous skeletal primordium of the limb. Joints appear as discrete regions of flattened cells that separate adjacent cartilaginous elements (Figure 2A). Initially, the joint interzones are distinguishable by the flattened morphology and closely packaging of the cells, which contrast with the rounded shape of the differentiating chondrocytes. In addition, the presence of gene expression domains of various markers, such as Gdf5 or Wnt14, makes the joint regions easily identifiable by in situ hybridization.^26^, ^27^ The initial formation of the joint interzone is later followed by differentiation of the synovial joint tissues, including the elaboration of the joint cavity.^28^ Surprisingly, cell death is a constant feature of the first step of joint formation (Figure 2A‐B) but appears absent or very reduced in the process of cavitation.^28^, ^29^, ^30^ Both in mammals (mouse) and birds (chick), the position of the future phalangeal joints in the autopod is preceded by the appearance of the row of dying cells (Figure 2A‐B).^31^, ^32^ This initial death process precedes and is unrelated to the formation of the joint cavity.^28^ Although experimental studies are scarce, the significance of this process may be related to provide the conditions required for local differentiation of the joint tissue precursors or to facilitate the arrival of specific joint precursors.^33^, ^34^ In contrast to the specification of the position of joints within the skeletal cartilaginous primordia, the process of cavitation appears to be due to cell and extracellular matrix rearrangement, with poor evidence for cell death.^35^

## 5 CELL DEATH DELINEATES THE MUSCLE BELLIES AND THE MYOTENDINOUS JUNCTIONS

The formation of a precise muscle pattern in the limb is a critical developmental event responsible for conferring specific biomechanical functions in each species of major evolutionary significance. The limb musculature develops from myogenic precursors that migrate from the somites into the limb bud. These myogenic cells invade the early limb primordium and form prominent premuscle masses along the dorsal and ventral surface of the differentiating skeletal primordia. The premuscle masses next become segregated by the intercalation of tendinous laminas at the level of formation of the main joints of the limb into the three muscle regions: the stylopodial (arm/thigh), the zeugopodial (forearm/leg), and autopodial (hand/foot). This initial process is next followed by a progressive splitting of the muscle bellies. The segregation of the muscle bellies is associated with their connection to their specific tendon blastema that, in turn, establish their attachment to the corresponding skeletal target.^36^

Although the elimination of the tail musculature during metamorphosis of amphibian anura has long been recognized a characteristic model of developmental cell death, muscle remodeling in developing vertebrate limbs has received little attention. In human fetuses, cell death has been functionally associated with myofiber differentiation and innervation of hand and tight muscles.^37^, ^38^ In avian embryos, muscle cell death has been analyzed in detail during the formation of muscle bellies of the foot.^39^ Notably, cell death is a central event that sculpts the muscle bellies (Figure 2C,D) and adjust the number of fibers that bind the tendon at the myotendinous junction complexes (Figure 2E,F). Similar to the establishment of neuronal connections, myogenic cells appear to receive survival signals from their target tendons that adjust the size and fibrillary composition of each particular muscle belly.

## 6 CELL DEATH IN THE EMBRYONIC LIMB ECTODERM DIRECTS LIMB OUTGROWTH

The early limb bud is a simple structure constituted by a core of mesodermal cells, first composed of skeletogenic precursors only, but the bud is very soon colonized by myogenic cells of somitic origin (see above). The ectoderm covers the surface of the bud and is continuous with the ectodermal surface of the embryonic body. From a functional point of view, the ectoderm, far from being a passive structure, is a major regulator of limb outgrowth. In the distal margin of the bud, the ectoderm appears thickened, forming the so‐called apical ectodermal ridge (AER), which provides signals that direct the proliferation of the subjacent mesoderm.^40^ AER integrity is essential to maintain outgrowth of the limb primordium and its flattening and degeneration once the digit primordia are formed mark the end of limb morphogenetic outgrowth. The function of the AER is mediated via the production and delivery of growth factors, including FGFs, BMPs, or Wnts, but FGFs (FGF4, and FGF8) are essential.^41^ Hence, surgical AER removal in the embryonic limb arrests limb outgrowth and induces cell death in the subjacent mesoderm resulting in truncation of the limb at a proximo‐distal level that is stage‐dependent.^42^ Importantly, local application of a source of the above‐mentioned FGFs can functionally replace the AER when this structure is surgically removed.^40^ Consistent with its growth‐promoting function, the AER is a transient structure that disappears when the most distal skeletal elements of the limb (distal phalanxes) are determined.^43^ As could be expected, the disappearance of the AER at the end of limb morphogenesis is mediated by cell death.^44^

In avian embryos, a widespread distribution of dead cells through the functionally‐active AER has been reported that does not result in its disappearance (Figure 1A‐B).^45^ The intensity of cell death shows differences between the wing and the leg buds that correlate with differences in the perimeter length of their AER. This observation suggests that cell death contributes to adapting the size and function of the AER which explains differences in limb bud morphology.

In mammalian embryos, ectodermal cell death appears focalized in the anterior and posterior margins of the bud, receiving the names of “foyer preaxial” and “foyer postaxial” respectively.^7^, ^46^ However, its function is also associated with an asymmetric pattern of growth of the limb bud. In fact, inhibition of the cell death of the “*foyer preaxial*” is observed in mouse mutants showing hyperphalangy of the first digit or preaxial polydactyly.^46^

## 7 CONTROL AND BIOLOGICAL SIGNIFICANCE OF CELL DEATH

The above‐described observations show that cell death accompanies all the developmental events occurring during limb formation. In some fashion, cell death should be considered equivalent to other cellular processes associated with embryonic development, such as cell proliferation, cell migration, tissue differentiation, and extracellular matrix deposition. However, the precise temporal and spatial distribution of most death processes and their different patterns in species with distinctive limb morphologies have often been suggested to indicate a specific regulation at the genetic level, leading to the term “programmed cell death”.

Initial studies analyzing the determination of the areas of cell death in the developing limb provided major support for the “programmed cell death” hypothesis.^47^ These studies showed that the prospective PNZ cells of the embryonic chick isolated from donor embryos and explanted to organ culture conditions underwent cell death on schedule when the donor embryo reached the stage when cell death occurs physiologically. These researchers proposed the existence of an intrinsic “death clock” in the prospective dying cells with important functions in limb morphogenesis. However, subsequent studies also performed in the avian limb showed that cell death is not rigidly determined in the PNZ.^48^, ^49^

**FIGURE 3:**
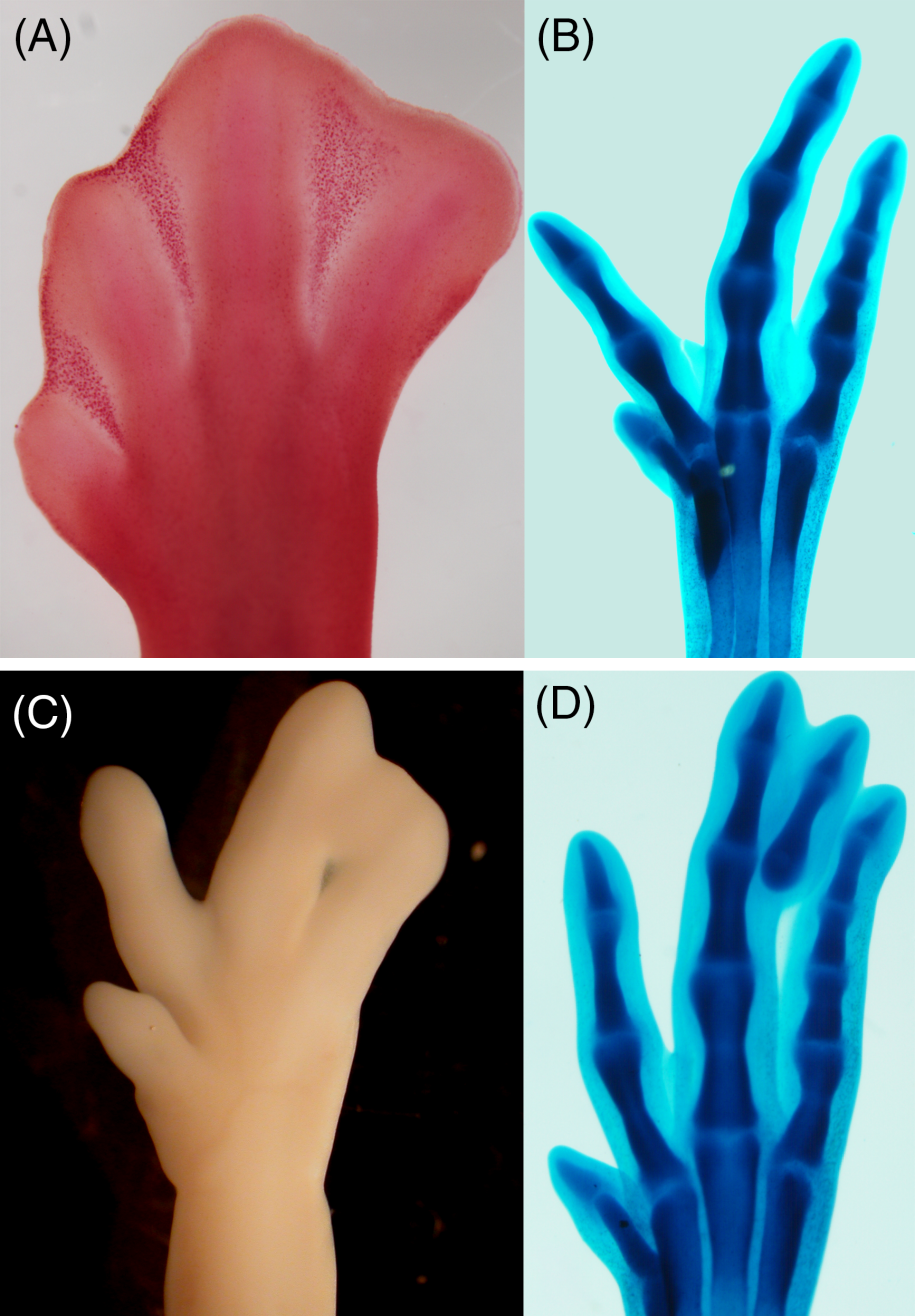
Developmental potential of the interdigital cells prior to cell death. A‐B, Neutral red staining showing the pattern of interdigital cell death in the INZs at stage 32, A, and the subsequent skeletal phenotype (alcian blue staining in panel B) in wild type chick embryos at stage 35. C‐D, The inhibition of cell death might result in membranous syndactyly (C; stage 34) or in the formation of an ectopic digit (D; stage 35). INZs, interdigital necrotic zones

The lack of a precocious death commitment of the cells located within the future dying domains has been best demonstrated for the INZs (Figure 3A‐D). Interdigital mesodermal cells are highly chondrogenic up to a few hours before the initiation of physiological degeneration.^50^, ^51^ This chondrogenic potential was fully confirmed by experiments in vivo, consisting of local application of microbeads uploaded with members of the TGF beta superfamily signaling via Smad 2/3 (Tgfbs and Activins).^52^, ^53^ A few hours after this treatment, the interdigital mesoderm formed a small ectopic cartilage.^52^ Three aspects of these experiments are notable: (a) the ectopic cartilage induced in the interdigit after the treatment maintained progressive outgrowth and formed a full digit with interphalangeal joints and associated tendons (Figure 3D); (b) the formation of the ectopic digits was not preceded by the regulation of important transcription factors in limb morphogenesis, such as Msx and Hoxd genes^51^, ^54^; and (c) the interdigits of the embryonic duck leg (fated to form a permanent interdigital web), despite having much larger dimensions than those of the chick, formed very small ectopic digits in in vivo experiments.^55^ These findings suggest that, in terms of programming, the interdigital cells of the chick are undifferentiated skeletal progenitors that die because of the local absence of differentiation and/or survival signals in a critical period of development. In the case of the duck, it is most likely that at the time of the treatments, many interdigital cells have initiated differentiation to form permanent membranous connective tissue, losing the ability to respond to chondrogenic stimuli. In fact, interdigital cell death in the duck is potentiated 24 hours after transient interdigital administration of FGFs.^56^ These growth factors are secreted by the AER and maintain subjacent mesoderm undifferentiated and proliferating, thus ensuring the outgrowth of the limb bud.^40^

The differentiation potential and plasticity of prospective dying cells in other areas of cell death of the embryonic limb have received little attention. However, it has been shown that the formation of distinct muscle bellies in the autopod is also a plastic process closely related to the formation of their corresponding skeletal and tendinous elements.^57^ Notably, the segmentation of autopodial premuscle masses involves an intense death process.^39^

In summary, the embryonic strategy used to build the different structural components of the limb can be divided into two periods: first, the formation of an excess mass of progenitors, which then, based on the differentiation signals, is segregated into a central component destined for to form the adult organ in question, and a peripheral population of progenitors that acts as a scaffold and is eliminated when it is no longer necessary to support the developing organ. Considering that interdigits do not contribute skeletal progenitors to digit rays, the reported truncation of the digits after surgical elimination of the interdigital tissue^58^ may illustrate this proposed structural scaffold function.

## 8 GENETIC ALTERATIONS AND SYNDACTYLY

The failure of interdigital cell death results in the permanence of interdigital tissue forming a membranous web (“*membranous syndactyly”*; Figure 3C) or the formation of extra skeletal tissue that joins adjacent digits in a variable range (“*bony syndactyly*”; Figure 3D). In humans, syndactyly is one of the most prevalent malformations,^59^ and many genetic alterations, both in humans and in laboratory animals, show variable levels of syndactyly. These observations, together with the diversity of the webbing in different tetrapods,^60^ have often led researchers to suspect the existence of a specific genetic regulation for INZ. However, the functional variety of genes whose mutations are associated with syndactyly and the heterogeneity of the phenotypes present in individuals with the same mutation^59^ make it difficult to identify a specific upstream genetic regulatory pathway for the degenerative process.

Genetic targets associated with syndactyly reflect the regulatory axis that globally controls limb development, but, with the exception of BMP signaling (see below), their function in interdigit remodeling lacks death specificity. Different levels of syndactyly accompanied dysregulation of each of the different steps that regulate limb outgrowth and tissue differentiation, including, (a) alterations in transcription factors modulating growth and skeletal tissue differentiation; (b) alterations in cell adhesion and extracellular matrix components; (c) dysregulation of the signaling pathways active in the interdigits; and (d) alteration of the cell degradation machinery.^61^

1. Numerous transcription factors are expressed in the interdigital regions, but none of them exhibit an exclusive expression in areas of cell death. Yet syndactyly is often associated with mutations of transcription factors. Among these mutations are single KO of HoxD13,^62^, ^63^ Bhlha9,^64^ Fingerin^65^ or compound KO of Msx1 and Msx2 genes.^66^ Syndactyly is even observed after dysregulation of genes not expressed in the interdigits such as N‐Myc.^67^ However, those mutations do not reflect a direct influence over a hypothetical death program. In most cases the syndactyly is syndromic, and the phenotype of the mutant embryos is caused by dysregulation of growth, cell differentiation, changes in the extracellular matrix, or transcriptional dysregulation of receptors and members of the cascade signaling implicated in most processes of limb morphogenesis (see^68^ as an illustrative example).
2. Knockdown of genes associated with cell adhesion, and/or with extracellular matrix processing often shows syndactyly. Among the genes are included integrins alpha 3 and 6,^69^ laminin alpha5,^70^ fibrillin 2,^71^ fibulin 1,^72^ nidogen 1 and 2,^73^ ADAMTS 5,9,20 metalloproteases^74^ and Fras1‐related extracellular matrix gene 1.^75^ Again, the basis for these syndactylies does not appear to be due to a direct effect on a hypothetical death program. Alterations are associated with dysregulation of tissue interactions and/or with the distribution of local signaling molecules.^71^, ^73^, ^74^
3. Growth and differentiation of limb tissue components is finely regulated by a network of locally produced extracellular signaling molecules. These ligands bind specific receptors and trigger intracellular cascades that direct proliferation, production of extracellular matrix, cell differentiation, and even cell death.^76^ Syndactylies associated with major signaling pathways are often explained not by the absence or abnormal presence of a specific factor but by the balance between distinct factors.
  BMPs (BMP 2, 4, 5, and 7) promote intense growth and differentiation of the limb prechondrogenic aggregates, but, at the same time, constitute the only factors with a demonstrated direct effect promoting cell death in undifferentiated limb skeletal progenitors.^77^, ^78^ These functionally contradictory effects can be explained by two complementary mechanisms. On one side, the pro‐apoptotic influence of local BMPs may be attenuated by a coincident expression of BMP antagonists.^79^ Consistent with this interpretation, the BMP antagonist *Gremlin 1* is a recognized marker of the interdigits in species with webbed digits.^18^, ^60^, ^80^ On the other side, the pro‐condrogenic influence of BMPs over the skeletal progenitors is dependent on the expression of SOX9. SOX9 is a master gene of chondrogenesis that modifies the configuration of chromatin promoting the expression of downstream chondrogenic genes.^81^ In the absence of SOX9 skeletal progenitors undergo cell death when exposed to BMPs^82^, ^83^ suggesting the implication of chromatin organization in the activation of the death program. In fact, the function of SOX9 is critically modulated by cofactors via chromatin modifications.^81^, ^84^
  The proapoptotic function of BMPs is reinforced by locally produced retinoic acid metabolites^85^, ^86^, ^87^ that also downregulate FGF gene expression. In turn, FGF signaling, represented mainly by FGF4 and FGF8, is a potent survival pathway, that protects progenitors from death but also maintains progenitors in an undifferentiated state that makes them sensitive to BMP‐mediated death signals.^56^, ^88^ The function of FGFs, as growth and anti‐apoptotic signals, is also shared by distinct members of the Wnt family.^89^ Insulin‐like growth factors^90^ and Notch signaling, acting at the ectodermal levels have also been implicated in the network that governs limb growth and morphogenesis.^91^ Finally, Hedgehog signaling critically influences the distribution of other signaling pathways.
  Consistent with the involvement of all these signaling pathways in the course of digit specification and morphogenesis a variable pattern of syndactyly often accompanies single or compound mutations in members of the mentioned signaling cascades including BMP signaling,^78^, ^92^, ^93^, ^94^, ^95^ FGF signaling,^96^, ^97^ Wnt signaling,^89^, ^98^, ^99^, ^100^ Hedgehog signaling,^101^, ^102^ retinoic acid signaling,^103^, ^104^ Notch signaling,^91^ and TGFbeta signaling.^105^ Notably, combinations of knockdown of an antagonist of BMPs such as Noggin with overexpression of Indian hedgehog (Ihh) result in intense syndactyly, which illustrates the importance of maintaining an appropriate signal equilibrium rather than acting separately.^106^
4. Dysregulation of the cell destructive machinery: It could be though that most syndactylies are due to disruption of the cell degradation machinery. However, this is not the case. Syndactylies caused by this mechanism are not spontaneously observed in humans and their experimental induction requires silencing of various components of the degradative machinery.
  Apoptosis dependent on caspases activated through the so‐called intrinsic or mitochondrial pathway is a prominent feature in the INZ,^107^ but the involvement of the extrinsic pathway, cannot be discarded.^108^, ^109^ However, genetic and pharmacological inhibition of caspases did not cause syndactyly.^110^ In addition, single gene silencing of members of the *Bcl2* gene family implicated in caspase activation via mitochondrial permeabilization, such as *Bak* or *Bax*,^111^ does not cause syndactyly. In contrast, compound gene silencing of various pro‐apoptotic members of the *Bcl2* gene family shows a variable penetrance of syndactyly or delayed interdigit regression.^111^, ^112^, ^113^, ^114^ In these cases, the syndactylous phenotype may be reinforced by blocking lysosomal activation and permeability.^115^ Members of the pro‐apoptotic gene family, such as BAK, permeabilize not only the mitochondrial membrane but also the lysosomal membranes. Therefore, their silencing may also reflect the pro‐death function of lysosomes.^116^, ^117^ In fact, syndactyly by compound gene silencing of *Bak* and *Bax*, is more accentuated when combined with silencing of the autophagic regulator *Atg5*.^111^ This finding indicates that, double knockdown of *Bak* and *Bax* does not fully inhibit cell death of all prospective dying interdigital progenitors.
  As mentioned above, lysosomes are also major players during interdigit remodeling^118^, ^119^ and autophagy is detectable in INZ cells.^111^, ^119^ Lysosomes appears to reflect an important degenerative pathway of adult and embryonic cells that has been termed cell senescence.^120^, ^121^ Cell senescence, is a prominent feature during interdigit remodeling.^122^, ^123^ This process is characterized by proliferation arrest, lysosomal hypertrophy, and activation of a secretory phenotype, that reinforces and spread degeneration. Lysosomal activation is best detectable by histochemical detection of β‐galactosidase at pH 6, but includes up‐regulation of most lysosomal enzymes^124^ including the activation of autophagy.^125^ However, no syndactylous phenotypes have been associated with dysregulation of lysosomal activity, either via cell senescence, autolysis, or autophagy, except for a minor delay in interdigit regression after *Atg5* gene silencing.^111^
  Together, these findings indicate that syndactyly reflects a dysregulation of redundant cell self‐destruction machinery rather than uncovering the genetic control of interdigit remodeling.

## 9 CONCLUDING REMARKS

In embryonic vertebrates, cell death is a relevant cell behavior that coordinates with other cell processes to form a limb with a precise shape and structural organization. There are two main developmental processes characterized by well‐defined areas of cell death: changes in the symmetry of the growing limb bud and tissue differentiation of the different tissue components of the musculoskeletal system. Furthermore, changes in the pattern of cell death among distinct vertebrate species contribute to the formation of specialized limbs adapted to serve very distinct functions, such as flying in birds or swimming in aquatic mammals.^126^ Considering this evidence, it cannot be questioned whether cell death is developmentally programmed. However, programming can be established at different levels. The experimental data surveyed here do not support the occurrence of specific upstream master transcription regulators that predetermine cells to die. In contrast, most observations indicate that specification of the dying areas is instructed by local signals within the limb bud. The coincident expression domains of BMP genes and the areas of cell death, together with the death inducing effect of BMPs provides a genetic basis explaining the position of the areas of cell death within the limb primordium. However, at mechanistic level cell death appears dependent on the characters of the target cells. This interpretation is consistent with a role of epigenetic factors accounting for the dual outcome of skeletal progenitors to differentiate or to die in response to common signals, proposed recently.^127^, ^128^

## AUTHOR CONTRIBUTIONS

**Juan Montero:** Conceptualization; investigation; methodology; project administration; resources; supervision; validation; visualization; writing‐original draft; writing‐review and editing. **Carlos Lorda‐Diez:** Conceptualization; investigation; methodology; resources; supervision; validation; visualization; writing‐original draft; writing‐review and editing. **Cristina Sanchez‐Fernandez:** Conceptualization; formal analysis; investigation; methodology; resources; supervision; validation; visualization; writing‐original draft. **Juan Hurle:** Conceptualization; formal analysis; funding acquisition; investigation; methodology; project administration; resources; supervision; validation; visualization; writing‐original draft; writing‐review and editing.

## References

[1] Glücksmann A. Cell deaths in normal vertebrate ontogeny. Biol Rev Camb Phil Soc. 1951;26:59‐86. 10.1111/j.1469-185x.1951.tb00774.x 24540363

[2] Kerr JF, Wyllie AH, Currie AR. Apoptosis: a basic biological phenomenon with wide‐ranging implications in tissue kinetics. Br J Cancer. 1972;26:239‐257. 4561027 10.1038/bjc.1972.33 PMC2008650

[3] Ellis HM, Horvitz HR. Genetic control of programmed cell death in the nematode *C. elegans* . Cell. 1986;44:817‐829. 3955651 10.1016/0092-8674(86)90004-8

[4] Fuchs Y, Steller H. Programmed cell death in animal development and disease. Cell. 2011;147:742‐758. 22078876 10.1016/j.cell.2011.10.033 PMC4511103

[5] Montero JA, Hurlé JM. Sculpturing digit shape by cell death. Apoptosis. 2010;15:365‐375. 20041300 10.1007/s10495-009-0444-5

[6] Montero JA, Sanchez‐Fernandez C, Lorda‐Diez CI, Garcia‐Porrero JA, Hurle JM. DNA damage precedes apoptosis during the regression of the interdigital tissue in vertebrate embryos. Sci Rep. 2016;6:35478. 27752097 10.1038/srep35478 PMC5067507

[7] Milaire J. A new interpretation of the necrotic changes occurring in the developing limb bud paddle of mouse embryos based upon recent observations in four different phenotypes. Int J Dev Biol. 1992;36:169‐178. 1627467

[8] Fernández‐Terán MA, Hinchliffe JR, Ros MA. Birth and death of cells in limb development: a mapping study. Dev Dyn. 2006;235:2521‐2537. 16881063 10.1002/dvdy.20916

[9] Cameron JA, Fallon JF. The absence of cell death during development of free digits in amphibians. Dev Biol. 1977;55:331‐338. 838123 10.1016/0012-1606(77)90176-2

[10] Vlaskalin T, Wong CJ, Tsilfidis C. Growth and apoptosis during larval forelimb development and adult forelimb regeneration in the newt (*Notophthalmus viridescens*). Dev Genes Evol. 2004;214:423‐431. 15322877 10.1007/s00427-004-0417-1

[11] Shimizu‐Nishikawa K, Nishimatsu S, Nishikawa A. Strategies to detect interdigital cell death in the frog, *Xenopus laevis*: T3 accerelation, BMP application, and mesenchymal cell cultivation. In Vitro Cell Dev Biol Anim. 2012;48:313‐325. 22580907 10.1007/s11626-012-9508-x

[12] Franssen RA, Marks S, Wake D, Shubin N. Limb chondrogenesis of the seepage salamander, *Desmognathus aeneus* (amphibia: plethodontidae). J Morphol. 2005;265:87‐101. 15880507 10.1002/jmor.10339

[13] Hinchliffe JR, Ede DE. Limb development in the polydactylous talpid3 mutant of the fowl. J Embryol Exp Morph. 1967;17:385‐404.

[14] Hinchliffe JR, Ede DE. Cell death and the de‐ velopment of limb form and skeletal pattern in normal and wingless (ws) chick embryos. J Embryol Exp Morph. 1973;30:753‐772. 4272514

[15] Zakeri Z, Quaglino D, Ahuja HS. Apoptotic cell death in the mouse limb and its suppression in the hammertoe mutant. Dev Biol. 1994;165:294‐297. 8088447 10.1006/dbio.1994.1255

[16] Knudsen TB, Kochhar DM. The role of morphogenetic cell death during abnormal limb‐bud outgrowth in mice heterozygous for the dominant mutation Hemimelia‐extra toe (Hmx). J Embryol Exp Morphol. 1981;65(Suppl):289‐307. 7334311

[17] Fallon JF, Cameron J. Interdigital cell death during limb development of the turtle and lizard with an interpretation of evolutionary significance. J Embryol Exp Morphol. 1977;40:285‐289. 915430

[18] Weatherbee SD, Behringer RR, Rasweiler JJ 4th, Niswander LA. Interdigital webbing retention in bat wings illustrates genetic changes underlying amniote limb diversification. Proc Natl Acad Sci U S A. 2006;103:15103‐15107. 17015842 10.1073/pnas.0604934103 PMC1622783

[19] Hurle JM, Garcia‐Martinez V, Gañan Y, Climent V, Blasco M. Morphogenesis of the prehensile autopodium in the common chameleon (Chamaeleo chamaeleo). J Morphol. 1987;194:187‐194. 29914225 10.1002/jmor.1051940207

[20] Diaz RE Jr, Trainor PA. Hand/foot splitting and the ‘re‐evolution’ of mesopodial skeletal elements during the evolution and radiation of chameleons. BMC Evol Biol. 2015;15:184. 26382964 10.1186/s12862-015-0464-4 PMC4574539

[21] Cooper KL, Sears KE, Uygur A, et al. Patterning and post‐patterning modes of evolutionary digit loss in mammals. Nature. 2014;511(7507):41‐45. 24990742 10.1038/nature13496 PMC4228958

[22] Tosney KW, Schroeter S, Pokrzywinski JA. Cell death delineates axon pathways in the hindlimb and does so independently of neurite outgrowth. Dev Biol. 1988;130:558‐572. 3197924 10.1016/0012-1606(88)90351-x

[23] Zuzarte‐Luis V, Montero JA, Torre‐Perez N, Garcia‐Porrero JA, Hurle JM. Cathepsin D gene expression outlines the areas of physiological cell death during embryonic development. Dev Dyn. 2007;236:880‐885. 17260350 10.1002/dvdy.21076

[24] Noro M, Yuguchi H, Sato T, et al. Role of paraxial mesoderm in limb/flank regionalization of the trunk lateral plate. Dev Dyn. 2011;240:1639‐1649. 21608076 10.1002/dvdy.22666

[25] Cohn MJ, Izpisua‐Belmonte JC, Abud H, Heath JK, Tickle C. Fibroblast growth factors induce additional limb development from the flank of chick embryos. Cell. 1995;80:739‐746. 7889567 10.1016/0092-8674(95)90352-6

[26] Merino R, Macias D, Gañan Y, et al. Expression and function of Gdf‐5 during digit skeletogenesis in the embryonic chick leg bud. Dev Biol. 1999;206:33‐45. 9918693 10.1006/dbio.1998.9129

[27] Hartmann C, Tabin CJ. Wnt‐14 plays a pivotal role in inducing synovial joint formation in the developing appendicular skeleton. Cell. 2001;104:341‐351. 11239392 10.1016/s0092-8674(01)00222-7

[28] Pitsillides AA, Ashhurst DE. A critical evaluation of specific aspects of joint development. Dev Dyn. 2008;237:2284‐2294. 18729226 10.1002/dvdy.21654

[29] Mitrovic DR. Development of the metatarsophalangeal joint of the chick embryo: morphological, ultrastructural and histochemical studies. Am J Anat. 1977;150:333‐348. 920633 10.1002/aja.1001500207

[30] Ito MM, Kida MY. Morphological and biochemical re‐evaluation of the process of cavitation in the rat knee joint: cellular and cell strata alterations in the interzone. J Anat. 2000;197(Pt 4):659‐679. 11197539 10.1046/j.1469-7580.2000.19740659.x PMC1468181

[31] Mori C, Nakamura N, Kimura S, Irie H, Takigawa T, Shiota K. Programmed cell death in the interdigital tissue of the fetal mouse limb is apoptosis with DNA fragmentation. Anat Rec. 1995;242:103‐110. 7604973 10.1002/ar.1092420114

[32] Kimura S, Shiota K. Sequential changes of programmed cell death in developing fetal mouse limbs and its possible roles in limb morphogenesis. J Morphol. 1996;229:337‐346. 8765811 10.1002/(SICI)1097-4687(199609)229:3<337::AID-JMOR8>3.0.CO;2-V

[33] Ray A, Singh PN, Sohaskey ML, Harland RM, Bandyopadhyay A. Precise spatial restriction of BMP signaling is essential for articular cartilage differentiation. Development. 2015;142:1169‐1179. 25758226 10.1242/dev.110940 PMC4360183

[34] Shwartz Y, Viukov S, Krief S, Zelzer E. Joint development involves a continuous influx of Gdf5‐positive cells. Cell Rep. 2016;15:2577‐2587. 27292641 10.1016/j.celrep.2016.05.055 PMC4920976

[35] Pacifici M, Koyama E, Iwamoto M. Mechanisms of synovial joint and articular cartilage formation: recent advances, but many lingering mysteries. Birth Defects Res C Embryo Today. 2005;75:237‐248. 16187328 10.1002/bdrc.20050

[36] Kardon G. Muscle and tendon morphogenesis in the avian hind limb. Development. 1998;125:4019‐4032. 9735363 10.1242/dev.125.20.4019

[37] Fidziańska A, Goebel HH. Human ontogenesis. 3. Cell death in fetal muscle. Acta Neuropathol. 1991;81:572‐577. 1858485 10.1007/BF00310140

[38] Kim JH, Abe S, Shibata S, et al. Dense distribution of macrophages in flexor aspects of the hand and foot of mid‐term human fetuses. Anat Cell Biol. 2012;45:259‐267. 23301193 10.5115/acb.2012.45.4.259 PMC3531589

[39] Rodriguez‐Guzman M, Montero JA, Santesteban E, Gañan Y, Macias D, Hurle JM. Tendon‐muscle crosstalk controls muscle bellies morphogenesis, which is mediated by cell death and retinoic acid signaling. Dev Biol. 2007;302:267‐280. 17070795 10.1016/j.ydbio.2006.09.034

[40] Verheyden JM, Sun X. Embryology meets molecular biology: deciphering the apical ectodermal ridge. Dev Biol. 2017;429:387‐390. 28131856 10.1016/j.ydbio.2017.01.017 PMC5526742

[41] Rodriguez‐Leon J, Tomas AR, Johnson A, Kawakami Y. Recent advances in the study of limb development: the emergence and function of the apical ectodermal ridge. J Stem Cells. 2013;8:79‐98. 24698985

[42] Saunders JW Jr. The proximo‐distal sequence of origin of the parts of the chick wing and the role of the ectoderm. J Exp Zool. 1948;3:363‐403. 10.1002/jez.1401080304 18882505

[43] Gañan Y, Macias D, Basco RD, Merino R, Hurle JM. Morphological diversity of the avian foot is related with the pattern of msx gene expression in the developing autopod. Dev Biol. 1998;196:33‐41. 9527879 10.1006/dbio.1997.8843

[44] Kelley RO, Fallon JF. Ultrastructural analysis of the apical ectodermal ridge during vertebrate limb morphogenesis. 1. The human forelimb with special reference to gap junctions. Dev Biol. 1976;51:241‐256. 955259 10.1016/0012-1606(76)90141-x

[45] Todt WL, Fallon JF. Development of the apical ectodermal ridge in the chick leg bud and a comparison with the wing bud. Anat Rec. 1986;215:288‐304. 3740467 10.1002/ar.1092150312

[46] Milaire J, Rooze M. Hereditary and induced modifications of the normal necrotic patterns in the developing limb buds of the rat and mouse: facts and hipotheses. Arch Biol (Bruxelles). 1983;94:459‐490.

[47] Saunders JW Jr. Death in embryonic systems. Science. 1966;154:604‐612. 5332319 10.1126/science.154.3749.604

[48] Fallon JF, Saunders JW Jr. In vitro analysis of the control of cell death in a zone of prospective necrosis from the chick wing bud. Dev Biol. 1968;18:553‐570. 5710080 10.1016/0012-1606(68)90026-2

[49] Brewton RG, MacCabe JA. Ectodermal influence on physiological cell death in the posterior necrotic zone of the chick wing bud. Dev Biol. 1988;126:327‐330. 3350214 10.1016/0012-1606(88)90142-x

[50] Hurle JM, Gañan Y. Formation of extra‐digits induced by surgical removal of the apical ectodermal ridge of the chick embryo leg bud in the stages previous to the onset of interdigital cell death. Anat Embryol (Berl). 1987;176:393‐399. 3631538 10.1007/BF00310193

[51] Ros MA, Piedra ME, Fallon JF, Hurle JM. Morphogenetic potential of the chick leg interdigital mesoderm when diverted from the cell death program. Dev Dyn. 1997;208:406‐419. 9056644 10.1002/(SICI)1097-0177(199703)208:3<406::AID-AJA11>3.0.CO;2-Y

[52] Gañan Y, Macias D, Duterque‐Coquillaud M, Ros MA, Hurle JM. Role of TGF beta s and BMPs as signals controlling the position of the digits and the areas of interdigital cell death in the developing chick limb autopod. Development. 1996;122:2349‐2357. 8756280 10.1242/dev.122.8.2349

[53] Merino R, Macias D, Gañan Y, et al. Control of digit formation by activin signalling. Development. 1999;126:2161‐2170. 10207141 10.1242/dev.126.10.2161

[54] Ros MA, Macias D, Fallon JF, Hurle JM. Formation of extra digits in the interdigital spaces of the chick leg bud is not preceded by changes in the expression of the Msx and Hoxd genes. Anat Embryol (Berl). 1994;190:375‐382. 7840423 10.1007/BF00187295

[55] Macias D, Gañan Y, Hurle JM. Interdigital chondrogenesis and extra digit formation in the duck leg bud subjected to local ectoderm removal. Anat Embryol (Berl). 1992;186:27‐32. 1514701 10.1007/BF00710399

[56] Montero JA, Gañan Y, Macias D, et al. Role of FGFs in the control of programmed cell death during limb development. Development. 2001;128:2075‐2084. 11493529 10.1242/dev.128.11.2075

[57] Luxey M, Berki B, Heusermann W, Fischer S, Tschopp P. Development of the chick wing and leg neuromuscular systems and their plasticity in response to changes in digit numbers. Dev Biol. 2020;458:133‐140. 31697937 10.1016/j.ydbio.2019.10.035

[58] Dahn RD, Fallon JF. Interdigital regulation of digit identity and homeotic transformation by modulated BMP signaling. Science. 2000;289(5478):438‐441. 10903202 10.1126/science.289.5478.438

[59] Malik S. Syndactyly: phenotypes, genetics and current classification. Eur J Hum Genet. 2012;20:817‐824. 22333904 10.1038/ejhg.2012.14 PMC3400728

[60] Tokita M, Matsushita H, Asakura Y. Developmental mechanisms underlying webbed foot morphological diversity in Waterbirds. Sci Rep. 2020;10:8028. 32415088 10.1038/s41598-020-64786-8 PMC7229147

[61] Al‐Qattan MM. A review of the genetics and pathogenesis of syndactyly in humans and experimental animals: a 3‐step pathway of pathogenesis. Biomed Res Int. 2019;9652649. 31637260 10.1155/2019/9652649 PMC6766129

[62] Brison N, Tylzanowski P, Debeer P. Limb skeletal malformations—what the HOX is going on? Biomed Res Int. 2019;9652649. 10.1155/2019/9652649. 21782042

[63] Dai L, Liu D, Song M, et al. Mutations in the homeodomain of HOXD13 cause syndactyly type 1‐c in two Chinese families. PLoS One. 2014;9(5):e96192. 24789103 10.1371/journal.pone.0096192 PMC4006867

[64] Kataoka K, Matsushima T, Ito Y, Sato T, Shigetoshi S, Asahara H. Bhlha9 regulates apical ectodermal ridge formation during limb development. J Bone Miner Metab. 2018;36:64‐72. 28324176 10.1007/s00774-017-0820-0 PMC6324935

[65] Schatz O, Langer E, Ben‐Arie N. Gene dosage of the transcription factor Fingerin (bHLHA9) affects digit development and links syndactyly to ectrodactyly. Hum Mol Genet. 2014;23(20):5394‐5401. 24852374 10.1093/hmg/ddu257

[66] Lallemand Y, Nicola MA, Ramos C, Bach A, Saint Cloment C, Benoît R. Analysis of Msx1; Msx2 double mutants reveals multiple roles for Msx genes in limb development. Development. 2005;132:3003‐3014. 15930102 10.1242/dev.01877

[67] Ota S, Zhou ZQ, Keene DR, Knoepfler P, Hurlin PJ. Activities of N‐Myc in the developing limb link control of skeletal size with digit separation. Development. 2007;134:1583‐1592. 17360777 10.1242/dev.000703

[68] Kuss P, Villavicencio‐Lorini P, Witte F, et al. Mutant Hoxd13 induces extra digits in a mouse model of synpolydactyly directly and by decreasing retinoic acid synthesis. J Clin Invest. 2009;119:146‐156. 19075394 10.1172/JCI36851 PMC2613457

[69] De Arcangelis A, Mark M, Kreidberg J, Sorokin L, Georges‐Labouesse E. Synergistic activities of alpha3 and alpha6 integrins are required during apical ectodermal ridge formation and organogenesis in the mouse. Development. 1999;126:3957‐3968. 10433923 10.1242/dev.126.17.3957

[70] Miner JH, Cunningham J, Sanes JR. Roles for laminin in embryogenesis: exencephaly, syndactyly, and placentopathy in mice lacking the laminin alpha5 chain. J Cell Biol. 1998;143:1713‐1723. 9852162 10.1083/jcb.143.6.1713 PMC2132973

[71] Arteaga‐Solis E, Gayraud B, Lee SY, Shum L, Sakai L, Ramirez F. Regulation of limb patterning by extracellular microfibrils. J Cell Biol. 2001;154(2):275‐281. 11470817 10.1083/jcb.200105046 PMC2150751

[72] Debeer P, Schoenmakers E, Twal W, et al. The fibulin‐1 gene (FBLN1) is disrupted in a t(12;22) associated with a complex type of synpolydactyly. J Med Genet. 2002;39:98‐104. 11836357 10.1136/jmg.39.2.98 PMC1735038

[73] Bose K, Nischt R, Page A, Bader BL, Paulsson M, Smyth N. Loss of nidogen‐1 and ‐2 results in syndactyly and changes in limb development. J Biol Chem. 2006;281:39620‐39629. 17023412 10.1074/jbc.M607886200

[74] McCulloch DR, Nelson CM, Dixon LJ, et al. ADAMTS metalloproteases generate active versican fragments that regulate interdigital web regression. Dev Cell. 2009;17:687‐698. 19922873 10.1016/j.devcel.2009.09.008 PMC2780442

[75] Smyth I, Du X, Taylor MS, Justice MJ, Beutler B, Jackson J. The extracellular matrix gene Frem1 is essential for the normal adhesion of the embryonic epidermis. Proc Natl Acad Sci U S A. 2004;101:13560‐13565. 15345741 10.1073/pnas.0402760101 PMC518794

[76] Zuzarte‐Luis V, Hurlé JM. Programmed cell death in the developing limb. Int J Dev Biol. 2002;46:871‐876. 12455623

[77] Macias D, Gañan Y, Sampath TK, Piedra ME, Ros MA, Hurle JM. Role of BMP‐2 and OP‐1 (BMP‐7) in programmed cell death and skeletogenesis during chick limb development. Development. 1997;124:1109‐1117. 9102298 10.1242/dev.124.6.1109

[78] Bandyopadhyay A, Tsuji K, Cox K, Harfe BD, Rosen V, Tabin CJ. Genetic analysis of the roles of BMP2, BMP4, and BMP7 in limb patterning and skeletogenesis. PLoS Genet. 2006;2:e216. 17194222 10.1371/journal.pgen.0020216 PMC1713256

[79] Lorda‐Diez CI, Montero JA, Rodriguez‐Leon J, Garcia‐Porrero JA, Hurle JM. Expression and functional study of extracellular BMP antagonists during the morphogenesis of the digits and their associated connective tissues. PLoS One. 2013;8(4):e60423. 23573253 10.1371/journal.pone.0060423 PMC3616094

[80] Merino R, Rodriguez‐Leon J, Macias D, Gañan Y, Economides AN, Hurle JM. The BMP antagonist Gremlin regulates outgrowth, chondrogenesis and programmed cell death in the developing limb. Development. 1999;126:5515‐5522. 10556075 10.1242/dev.126.23.5515

[81] Furumatsu T, Tsuda M, Yoshida K, et al. Sox9 and p300 cooperatively regulate chromatin‐mediated transcription. J Biol Chem. 2005;280(42):35203‐35208. 16109717 10.1074/jbc.M502409200

[82] Chimal‐Monroy J, Rodriguez‐Leon J, Montero JA, et al. Analysis of the molecular cascade responsible for mesodermal limb chondrogenesis: Sox genes and BMP signaling. Dev Biol. 2003;257(2):292‐301. 12729559 10.1016/s0012-1606(03)00066-6

[83] Montero JA, Lorda‐Diez CI, Francisco‐Morcillo J, Chimal‐Monroy J, Garcia‐Porrero JA, Hurle JM. Sox9 expression in amniotes: species‐specific differences in the formation of digits. Front Cell Dev Biol. 2017;5:23. 28386540 10.3389/fcell.2017.00023 PMC5362607

[84] Hattori T, Coustry F, Stephens S, et al. Transcriptional regulation of chondrogenesis by coactivator Tip60 via chromatin association with Sox9 and Sox5. Nucleic Acids Res. 2008;36(9):3011‐3024. 18390577 10.1093/nar/gkn150 PMC2396410

[85] Rodriguez‐Leon J, Merino R, Macias D, Gañan Y, Santesteban E, Hurle JM. Retinoic acid regulates programmed cell death through BMP signalling. Nat Cell Biol. 1999;1:125‐126. 10559885 10.1038/10098

[86] Hernández‐Martínez R, Castro‐Obregón S, Covarrubias L. Progressive interdigital cell death: regulation by the antagonistic interaction between fibroblast growth factor 8 and retinoic acid. Development. 2009;136:3669‐3678. 19820185 10.1242/dev.041954

[87] Díaz‐Hernández ME, Rios‐Flores AJ, Abarca‐Buis RF, Bustamante M, Chimal‐Monroy J. Molecular control of interdigital cell death and cell differentiation by retinoic acid during digit development. J Dev Biol. 2014;2:138‐157.

[88] Mariani FV, Fernandez‐Teran M, Ros MA. Ectoderm‐mesoderm crosstalk in the embryonic limb: the role of fibroblast growth factor signaling. Dev Dyn. 2017;246:208‐216. 28002626 10.1002/dvdy.24480 PMC8262604

[89] Villacorte M, Suzuki K, Hayashi K, et al. Antagonistic crosstalk of Wnt/beta‐catenin/Bmp signaling within the apical ectodermal ridge (AER) regulates interdigit formation. Biochem Biophys Res Commun. 2010;391:1653‐1657. 20043884 10.1016/j.bbrc.2009.12.109 PMC2823811

[90] Allan GJ, Flint DJ, Patel K. Insulin‐like growth factor axis during embryonic development. Reproduction. 2001;122:31‐39. 11425327 10.1530/rep.0.1220031

[91] Pan Y, Liu Z, Shen J, Kopan R. Notch1 and 2 cooperate in limb ectoderm to receive an early Jagged2 signal regulating interdigital apoptosis. Dev Biol. 2005;286:472‐482. 16169548 10.1016/j.ydbio.2005.08.037

[92] Guha U, Gomes WA, Kobayashi T, Pestell RG, Kessler JA. In vivo evidence that BMP signaling is necessary for apoptosis in the mouse limb. Dev Biol. 2002;249:108‐120. 12217322 10.1006/dbio.2002.0752

[93] Wong YL, Behringer RR, Kwan KM. Smad1/Smad5 signaling in limb ectoderm functions redundantly and is required for interdigital programmed cell death. Dev Biol. 2012;363:247‐257. 22240098 10.1016/j.ydbio.2011.12.037 PMC5134426

[94] Okada I, Hamanoue H, Terada K, et al. SMOC1 is essential for ocular and limb development in humans and mice. Am J Hum Genet. 2011;88:30‐41. 21194678 10.1016/j.ajhg.2010.11.012 PMC3014372

[95] Kaltcheva MM, Anderson MJ, Harfe BD, Lewandoski M. BMPs are direct triggers of interdigital programmed cell death. Dev Biol. 2016;411:266‐276. 26826495 10.1016/j.ydbio.2015.12.016 PMC7028150

[96] Wilkie AO, Patey SJ, Kan SH, van den Ouweland AM, Hamel BC. FGFs, their receptors, and human limb malformations: clinical and molecular correlations. Am J Med Genet. 2002;112:266‐278. 12357470 10.1002/ajmg.10775

[97] Lu P, Minowada G, Martin GR. Increasing Fgf4 expression in the mouse limb bud causes polysyndactyly and rescues the skeletal defects that result from loss of Fgf8 function. Development. 2006;133:33‐42. 16308330 10.1242/dev.02172

[98] Simon‐Chazottes D, Tutois S, Kuehn M, et al. Mutations in the gene encoding the low‐density lipoprotein receptor LRP4 cause abnormal limb development in the mouse. Genomics. 2006;87:673‐677. 16517118 10.1016/j.ygeno.2006.01.007

[99] Morello R, Bertin TK, Schlaubitz S, et al. Brachy‐syndactyly caused by loss of Sfrp2 function. J Cell Physiol. 2008;217:127‐137. 18446812 10.1002/jcp.21483 PMC2677682

[100] Ikegawa M, Han H, Okamoto A, et al. Syndactyly and preaxial synpolydactyly in the single Sfrp2 deleted mutant mice. Dev Dyn. 2008;237:2506‐2517. 18729207 10.1002/dvdy.21655

[101] Klopocki E, Lohan S, Brancati F, et al. Copy‐number variations involving the IHH locus are associated with syndactyly and craniosynostosis. Am J Hum Genet. 2011;88:70‐75. 21167467 10.1016/j.ajhg.2010.11.006 PMC3014361

[102] Anderson E, Peluso S, Lettice LA, Hill RE. Human limb abnormalities caused by disruption of hedgehog signaling. Trends Genet. 2012;28(8):364‐373. 22534646 10.1016/j.tig.2012.03.012

[103] Dupé V, Ghyselinck NB, Thomazy V, et al. Essential roles of retinoic acid signaling in interdigital apoptosis and control of BMP‐7 expression in mouse autopods. Dev Biol. 1999;208:30‐43. 10075839 10.1006/dbio.1998.9176

[104] Cunningham TJ, Chatzi C, Sandell LL, Trainor PA, Duester G. Rdh10 mutants deficient in limb field retinoic acid signaling exhibit normal limb patterning but display interdigital webbing. Dev Dyn. 2011;240:1142‐1150. 21360789 10.1002/dvdy.22583 PMC3081420

[105] Dunker N, Schmitt K, Krieglstein K. TGF‐beta is required for programmed cell death in interdigital webs of the developing mouse limb. Mech Dev. 2002;113:111‐120. 11960699 10.1016/s0925-4773(02)00015-1

[106] Murgai A, Altmeyer S, Wiegand S, Tylzanowski P, Stricker S. Cooperation of BMP and IHH signaling in interdigital cell fate determination. PLoS One. 2018;13:e0197535. 29771958 10.1371/journal.pone.0197535 PMC5957397

[107] Zuzarte‐Luis V, Berciano MT, Lafarga M, Hurlé JM. Caspase redundancy and release of mitochondrial apoptotic factors characterize interdigital apoptosis. Apoptosis. 2006;11:701‐715. 16532376 10.1007/s10495-006-5481-8

[108] Wride MA, Lapchak PH, Sanders EJ. Distribution of TNF alpha‐like proteins correlates with some regions of programmed cell death in the chick embryo. Int J Dev Biol. 1994;38:673‐682. 7779688

[109] Svandova EB, Vesela B, Lesot H, Poliard A, Matalova E. Expression of Fas, FasL, caspase‐8 and other factors of the extrinsic apoptotic pathway during the onset of interdigital tissue elimination. Histochem Cell Biol. 2017;147:497‐510. 27709293 10.1007/s00418-016-1508-6

[110] Chautan M, Chazal G, Cecconi F, Gruss P, Golstein P. Interdigital cell death can occur through a necrotic and caspase‐independent pathway. Curr Biol. 1999;9:967‐970. 10508592 10.1016/s0960-9822(99)80425-4

[111] Arakawa S, Tsujioka M, Yoshida T, et al. Role of Atg5‐dependent cell death in the embryonic development of Bax/Bak double‐knockout mice. Cell Death Differ. 2017;24(9):1598‐1608. 28574506 10.1038/cdd.2017.84 PMC5563990

[112] Lindsten T, Ross AJ, King A, et al. The combined functions of proapoptotic Bcl‐2 family members bak and bax are essential for normal development of multiple tissues. Mol Cell. 2000;6:1389‐1399. 11163212 10.1016/s1097-2765(00)00136-2 PMC3057227

[113] Ke FFS, Vanyai HK, Cowan AD, et al. Embryogenesis and adult life in the absence of intrinsic apoptosis effectors BAX, BAK, and BOK. Cell. 2018;173:1217‐1230. 29775594 10.1016/j.cell.2018.04.036

[114] Ren D, Tu HC, Kim H, et al. BID, BIM, and PUMA are essential for activation of the BAX‐ and BAK‐dependent cell death program. Science. 2010;330:1390‐1393. 21127253 10.1126/science.1190217 PMC3163443

[115] Johansson AC, Appelqvist H, Nilsson C, Kågedal K, Roberg K, Öllinger K. Regulation of apoptosis‐associated lysosomal membrane permeabilization. Apoptosis. 2010;15:527‐540. 20077016 10.1007/s10495-009-0452-5 PMC2850995

[116] Repnik U, Stoka V, Turk V, Turk B. Lysosomes and lysosomal cathepsins in cell death. Biochim Biophys Acta. 1824;2012:22‐33. 10.1016/j.bbapap.2011.08.016 21914490

[117] Karch J, Schips TG, Maliken BD, et al. Autophagic cell death is dependent on lysosomal membrane permeability through Bax and Bak. Elife. 2017;6:e30543. 29148970 10.7554/eLife.30543 PMC5697932

[118] Zuzarte‐Luis V, Montero JA, Kawakami Y, Izpisua‐Belmonte JC, Hurle JM. Lysosomal cathepsins in embryonic programmed cell death. Dev Biol. 2007;301:205‐217. 16987511 10.1016/j.ydbio.2006.08.008

[119] Montero JA, Lorda‐Diez CI, Certal AC, et al. Coordinated and sequential activation of neutral and acidic DNases during interdigital cell death in the embryonic limb. Apoptosis. 2010;15:1197‐1210. 20614251 10.1007/s10495-010-0523-7

[120] Storer M, Mas A, Robert‐Moreno A, et al. Senescence is a developmental mechanism that contributes to embryonic growth and patterning. Cell. 2013;155(5):1119‐1130. 24238961 10.1016/j.cell.2013.10.041

[121] Muñoz‐Espín D, Cañamero M, Maraver A, et al. Programmed cell senescence during mammalian embryonic development. Cell. 2013;155:1104‐1118. 24238962 10.1016/j.cell.2013.10.019

[122] Vasey DB, Wolf CR, Brown K, Whitelaw CB. Spatial p21 expression profile in the mid‐term mouse embryo. Transgenic Res. 2011;20:23‐28. 20349273 10.1007/s11248-010-9385-6

[123] Lorda‐Diez CI, Garcia‐Riart B, Montero JA, Rodriguez‐León J, Garcia‐Porrero JA, Hurle JM. Apoptosis during embryonic tissue remodeling is accompanied by cell senescence. Aging (Albany NY). 2015;7:974‐985. 26568417 10.18632/aging.100844 PMC4694067

[124] Kurz DJ, Decary S, Hong Y, Erusalimski JD. Senescence‐associated β‐galactosidase reflects an increase in lysosomal mass during replicative ageing of human endothelial cells. J Cell Sci. 2000;113:3613‐3622. 11017877 10.1242/jcs.113.20.3613

[125] Young AR, Narita M, Ferreira M, et al. Autophagy mediates the mitotic senescence transition. Genes Dev. 2009;23(7):798‐803. 19279323 10.1101/gad.519709 PMC2666340

[126] Cooper LN, Sears KE, Armfield BA, Kala B, Hubler M, Thewissen JGM. Review and experimental evaluation of the embryonic development and evolutionary history of flipper development and hyperphalangy in dolphins (Cetacea: Mammalia). Genesis. 2018;56(1):e23076. 10.1002/dvg.23076 29068152

[127] Sanchez‐Fernandez C, Lorda‐Diez CI, García‐Porrero JA, Montero JA, Hurlé JM. UHRF genes regulate programmed interdigital tissue regression and chondrogenesis in the embryonic limb. Cell Death Dis. 2019;10:347. 31024001 10.1038/s41419-019-1575-4 PMC6484032

[128] Sanchez‐Fernandez C, Lorda‐Diez CI, Hurlé JM, Montero JA. The methylation status of the embryonic limb skeletal progenitors determines their cell fate in chicken. Commun Biol. 2020;3:283. 32504030 10.1038/s42003-020-1012-3 PMC7275052

